# Historical and contemporary stable isotope tracer approaches to studying mammalian protein metabolism

**DOI:** 10.1002/mas.21507

**Published:** 2016-05-16

**Authors:** Daniel James Wilkinson

**Affiliations:** ^1^ MRC‐ARUK Centre for Musculoskeletal Ageing Research, Clinical, Metabolic and Molecular Physiology University of Nottingham, Royal Derby Hospital Centre Derby United Kingdom

**Keywords:** stable isotopes, protein metabolism, mass spectrometry

## Abstract

Over a century ago, Frederick Soddy provided the first evidence for the existence of isotopes; elements that occupy the same position in the periodic table are essentially *chemicall*y identical but differ in mass due to a different number of neutrons within the atomic nucleus. Allied to the discovery of isotopes was the development of some of the first forms of mass spectrometers, driven forward by the Nobel laureates JJ Thomson and FW Aston, enabling the accurate separation, identification, and quantification of the relative abundance of these isotopes. As a result, within a few years, the number of known isotopes both stable and radioactive had greatly increased and there are now over 300 stable or radioisotopes presently known. Unknown at the time, however, was the potential utility of these isotopes within biological disciplines, it was soon discovered that these stable isotopes, particularly those of carbon (^13^C), nitrogen (^15^N), oxygen (^18^O), and hydrogen (^2^H) could be chemically introduced into organic compounds, such as fatty acids, amino acids, and sugars, and used to “trace” the metabolic fate of these compounds within biological systems. From this important breakthrough, the age of the isotope tracer was born. Over the following 80 yrs, stable isotopes would become a vital tool in not only the biological sciences, but also areas as diverse as forensics, geology, and art. This progress has been almost exclusively driven through the development of new and innovative mass spectrometry equipment from IRMS to GC‐MS to LC‐MS, which has allowed for the accurate quantitation of isotopic abundance within samples of complex matrices. This historical review details the development of stable isotope tracers as metabolic tools, with particular reference to their use in monitoring protein metabolism, highlighting the unique array of tools that are now available for the investigation of protein metabolism *in vivo* at a whole body down to a single protein level. Importantly, it will detail how this development has been closely aligned to the technological development within the area of mass spectrometry. Without the dedicated development provided by these mass spectrometrists over the past century, the use of stable isotope tracers within the field of protein metabolism would not be as widely applied as it is today, this relationship will no doubt continue to flourish in the future and stable isotope tracers will maintain their importance as a tool within the biological sciences for many years to come. © 2016 The Authors. *Mass Spectrometry Reviews* Published by Wiley Periodicals, Inc. Mass Spec Rev

## I. INTRODUCTION: WHAT IS A STABLE ISOTOPE TRACER?

Since their initial isolation and implementation in the 1930s, stable isotope tracers have provided the scientific community with great insight into the understanding of the metabolism underlying disease (Kao et al., 2011), aging (Cuthbertson et al., 2005), and genetic disorders (Mew, Yudkoff, & Tuchman, 2014). While predominantly used for studying biological systems, stable isotopes are not limited to this area, and today have a wide range of applications, such as, art, forensics, and geology (Fortunato, Ritter, & Fabian, 2005; Meier‐Augenstein et al., 2011; Menicucci, Matthews, & Spero, 2013). Stable isotopes are elements that occupy the same position in the periodic table, are essentially “*chemically and functionally identical*,” but differ in mass due to a different number of neutrons within the atomic nucleus. This difference in mass makes these isotopes analytically distinguishable from each other; therefore, if introduced into a system the metabolic fate of these isotopes can be “traced.”

While stable isotopes are generally considered to be “*identical*” in behavior to the more abundant endogenous isotopes, there are certain exceptions to this rule that should be noted. Highly deuterated molecules, for example, can impact the rates of enzymatic reactions, with early work showing that ^2^H_2_‐labeled succinic acid is dehydrogenated at a slower rate than the endogenous compound (Erlenmeyer, Schoenauer, & Süllmann, 1936), this was believed to be due in part to the more stable chemical bonds created by deuterium compared to hydrogen (Jones & Leatherdale, 1991). These effects seem to be most significant in mammals for deuterated compounds only however, with other isotopes such as ^13^Carbon showing limited effects even at relatively high doses (approaching 60%; Gregg et al., 1973), in contrast in plants the ^13^Carbon isotope will be handled differently based on whether the plant utilizes the C3 or C4 pathways for carbon fixation during photosynthesis (Hobbie & Werner, 2004). Despite these potential consequences, for the vast majority of the biological processes probed using stable isotopes, the amounts used, and hence levels of enrichment reached, are so low they will have no effect on normal metabolism. Therefore, by replacing one (or more) of the ^12^Carbon, ^14^Nitrogen, ^16^Oxygen, or ^1^Hydrogen (however not limited to these alone, see Table 1) within a compound of interest with their heavier less naturally abundant stable isotopic equivalents; ^13^Carbon (^13^C), ^15^Nitrogen (^15^N), ^18^Oxygen (^18^O), or ^2^Hydrogen (^2^H or D), respectively, a stable isotope tracer is created. These labeled compounds can then be introduced into a system to be studied, and the fate of this compound and/or its metabolites can be monitored over time providing a dynamic measurement of metabolism within this system. In most cases, the sensitivity of the analytical equipment available (e.g., mass spectrometry) ensures that these labeled compounds can be added to the system at comparatively low levels. As an example of the utility of these tracers, the measurement of amino acid and protein metabolism will briefly be described, while this example refers to protein metabolism in particular, with slight sampling and kinetic modeling modifications these techniques can be applied for the majority of substrate metabolism measures; from lipids, to sugars to DNA. In addition, before moving on, it should be highlighted that this review will concentrate solely on stable isotopes with only brief reference to radioisotopes, despite not being discussed in great detail here radioisotopes and their tracers continue to provide valuable insight into the regulation of metabolism and for those interested in learning more about these and other tracer techniques the reader is referred to the definitive text on isotope tracers by Wolfe & Chinkes (2005).

**Table 1:**
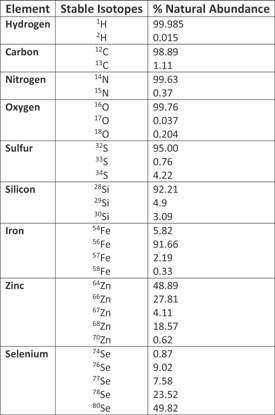
Summary of the more commonly used stable isotopes for tracer work including their natural abundance (adapted from Wolfe & Chinkes, 2005)

### A. Protein Turnover in Mammals: An Example of the Application of Stable Isotope Tracers

**Figure 1:**
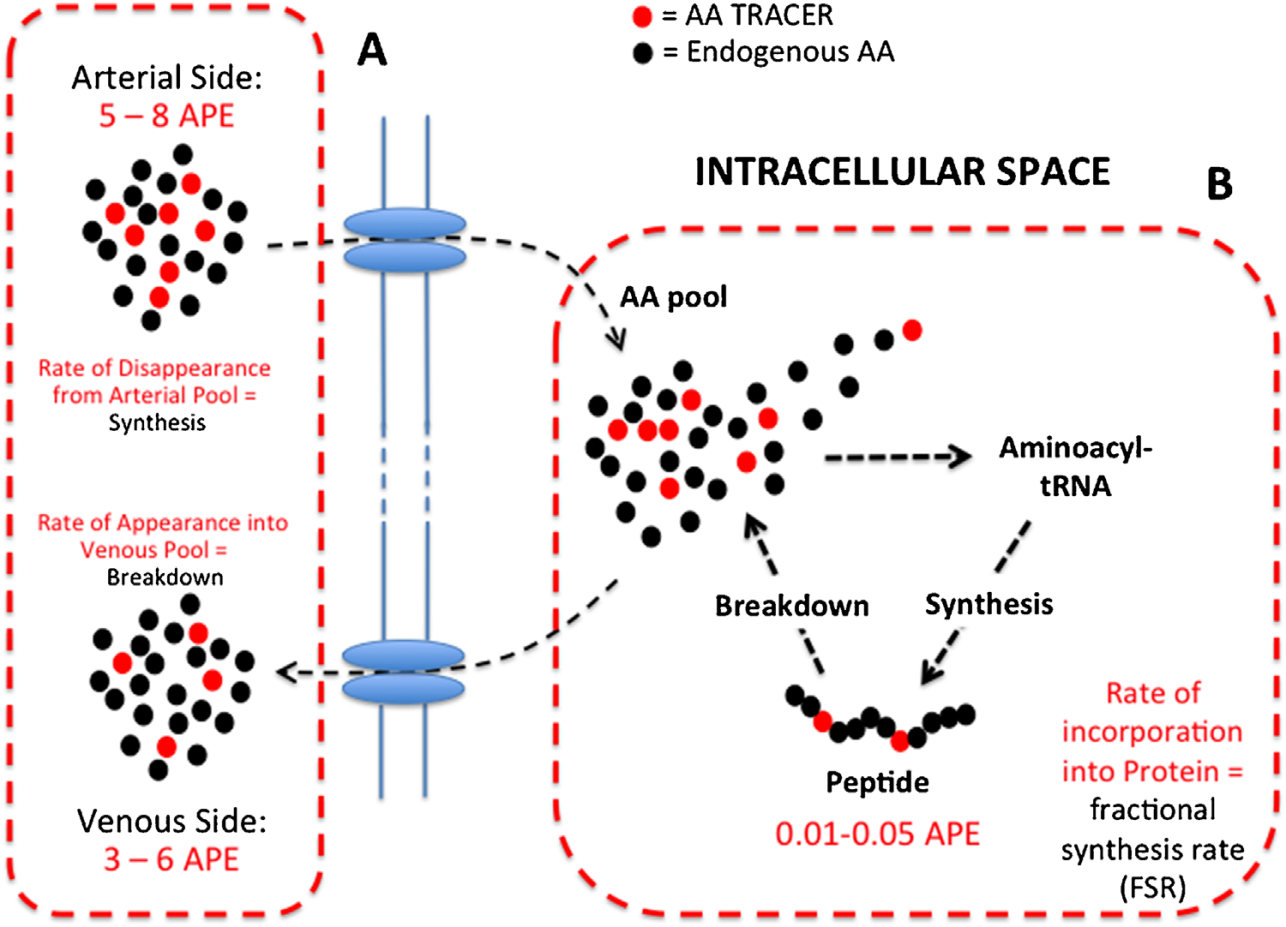
Schematic of how stable isotope tracers are used to measure protein turnover within a mammalian system highlighting: (**A**) Arterial‐Venous balance techniques and (**B**) FSR techniques (Reprinted with permission from Atherton et al., 2015 (Copyright 2015, Elsevier)).

Novel stable isotope tracer methodologies have been extensively developed over the past 80 years, and it is now possible to accurately measure changes in protein metabolism from the whole body down to a tissue‐specific level and even individual protein level with relative ease (Rennie, 1999). Figure 1 provides a typical schematic example of how stable isotope tracers are used in mammalian systems for the study of protein turnover. Typically, a labeled amino acid (e.g., 1,2 ^13^C_2_ leucine, ring D_5_ phenylalanine, or ^15^N lysine) is introduced into the system at a constant rate following a primed bolus (this is not always essential, single flooding and bolus introduction can be performed also; Garlick et al., 1989; Wilkinson et al., 2014), this helps ensure rapid equilibrium of the exogenously introduced tracer within all the endogenous tracee (amino acid; AA) pools, that is, arterial, venous, intracellular/extracellular spaces. The tracer is then taken up into the cell/tissue of interest at the same rate as the endogenous amino acid, the rate of the disappearance of the tracer from the arterial pool over time provides a proxy for synthesis, while the rate of appearance of the tracee, which dilutes the tracer in the venous pool, provides a proxy for breakdown; provided the AA being studied is not subject to secondary metabolism within the tissue, such as phenylalanine in muscle (although enzymatic conversion to tyrosine must also be considered with this AA when measuring whole body metabolism: Biolo et al., 1992a, 1995a, 1994). Therefore, just through the sampling of arterial and venous blood (arterial–venous [A–V] balance) across the tissue bed (e.g., muscle group), rates of protein turnover of that tissue can be determined with certain caveats (Biolo et al., 1994). Furthermore, when the tracer enters the intracellular AA pool, it will be sequestered by its corresponding acyl‐tRNA and incorporated into proteins within the tissue. By sampling these tissues through biopsy procedures, chemically isolating and hydrolyzing the protein of interest to its constituent amino acids, the amount of AA tracer incorporated into the protein can be measured, which with measurement of tracer enrichment in the precursor pool (the amino‐acyl‐tRNA), or a proxy surrogate (intracellular AA pool is normally used instead as it is easier to access and isolate than tRNA), means that a fractional synthesis rate can be determined (FSR; (Rennie et al., 1982). While both methods are valid for measuring protein turnover, the FSR method is considered the current “gold standard” as A–V balance measures can be significantly influenced by AA metabolism in other tissues (e.g., bone, skin, or limbs) and perturbations to blood flow, whereas the incorporation or FSR approach samples the specific tissue/protein of interest. As a result of these methods, a wealth of knowledge has been obtained regarding the regulation of protein turnover in different species and different physiological states, and is now an increasingly common tool in biological research. Using such tracer methodologies, we now know the tissue pools are continually under a constant state of turnover, with each tissue turning over at vastly differing rates dependent on their role in the body; for example, splanchnic tissues can turnover at rates of ∼50%/d, while skeletal muscle turns over much slower at around 1–1.5%/d; Halliday & McKeran, 1975; O'keefe et al., 2006), and is finely regulated during periods of feeding and fasting (Atherton & Smith, 2012). Moreover, due to the important metabolic contribution of this organ, tracer‐based studies involving skeletal muscle have dominated in recent years and regulation of its metabolism by nutrition, exercise, and hormones is now well understood, yet there is still much that remains to be uncovered.

During post‐absorptive/fasted phases of the day, the skeletal muscle protein pool is in a state of negative protein balance, that is, protein is broken down at a rate that is faster than the rate of the synthesis of new proteins, this is primarily driven through the need to provide a continuous supply of amino acids to the other vital organs including the rapidly turning over vital splanchnic organs (Biolo, Zhang, & Wolfe, 1995b). Following nutritional intake, this scenario is reversed with protein and amino acids providing the substrate for increasing synthesis of new proteins, while also inhibiting protein breakdown through elevations in insulin (Rennie et al., 1982; Wilkes et al., 2009). This creates a net positive protein balance, MPS > MPB, and protein lost from muscle during the post‐ absorptive phase is recovered. Thereby creating a state of dynamic equilibrium in muscle for maintenance of mass in healthy adult humans (Atherton & Smith, 2012).

Stable isotope tracers have also allowed us to investigate in greater detail the important dietary constituents driving this coordinated regulation of muscle protein metabolism. It was shown that feeding protein alone could recapitulate the fed state increases in MPS and inhibition of protein breakdown associated with a mixed meal, highlighting that protein *per se* is the primary driver behind the anabolic regulation of protein metabolism (Rennie et al., 1982). It was thereafter demonstrated that AA alone could provide the same effect (Bennet et al., 1989a), with essential AA (EAA) in particular key to this stimulation (Smith et al., 1992, 1998). Protein breakdown in contrast has been shown to be regulated through the release of insulin following feeding rather than through a direct effect of AA alone (although some AA are potent insulin secretagogues; Greenhaff et al., 2008). Furthermore, due to the availability of multiply labeled stable isotope tracers and the development of GC‐C & pyrolysis—IRMS (described in more detail later), more recent investigations have highlighted the temporal nature of this post feeding synthetic response, which begins 30 min post‐ingestion of AA substrate and peaks 90–120 min post‐feed (replacing fasting losses), after which protein synthesis returns to post‐absorptive rates and is refractory to further stimulation despite the presence of available AA substrate; a phenomenon termed “muscle full” (Atherton et al., 2010). Another important aspect of this nutritional response uncovered using tracers is the so called “anabolic blunting” associated with age, immobilization, and chronic metabolic disease. It has been shown on numerous occasions that older muscle is resistant or insensitive to the anabolic effect of protein/AA feeding (Brook et al., 2016). When provided with the same amount of nutrition as young healthy adults, the stimulatory protein synthetic response (and normal inhibition of protein breakdown) is significantly impaired in the old compared to the young (Cuthbertson et al., 2005; Wilkes et al., 2009). In addition, these techniques have demonstrated the existence of a “blunted” response of elderly muscle to resistance exercise, when compared to young (Kumar et al., 2009). This “metabolic blunting” has been proposed to play a critical role in the age related loss of muscle (sarcopenia), which occurs at ∼1–1.5%/yrs in over 50s, leading to a significant impact on muscle and metabolic health in old age (Mitchell et al., 2012). These aspects of metabolism still remain contentious, however, and are derived from studies performed over acute time periods (3–6 hr), with results assumed to extrapolate to chronic free‐living situations. This can be problematic, as highlighted recently by Mitchell et al., (2014), where acute measures of FSR collected over a few hours, did not correlate with muscle mass gains due to resistance exercise training (Mitchell et al., 2014), highlighting discordance between metabolic and physiological adaptations. The fact is that the homeostatic balance between synthesis and breakdown over days, weeks, months in response to normal living is still very difficult to measure, and any small change such as that provided by metabolic blunting may be very difficult to detect. However, this phenomenon has led to a great deal of interest in recent years into interventions to prevent, slow or delay this progressive decline, with the overarching aim of improving quality of life into older age. Without stable isotope tracer techniques, this understanding of the intricacies of changes to metabolism across the life span would not have been possible, and tracer techniques continue today to provide valuable biological insight. The remaining sections of this review will aim to highlight the development of some of the above stable isotope tracer techniques into the common research tools of today, from their initial identification and implementation to the methodological developments that drove these techniques forward.

## II. THE DISCOVERY OF STABLE ISOTOPES AND THE EARLY PIONEERS

Although the first use of the word isotope was not referred to until 1913 when Frederick Soddy conceived the word from the Greek *isos topos* meaning same place (Soddy, 1913a). The idea and/or conception of the presence of the “isotope” has a long history, extending up to a century before Soddy postulated the term. As eloquently stated in a historical review from this journal by Budzikiewicz & Grigsby (2006), some of the first theories on this matter evolved from the ideas put forward by William Prout in his anonymous writings on the weight of atoms, suggesting that all elementary numbers were seemingly multiples of hydrogen (Anonymous, 1815). It was becoming clearer toward the end of 19th Century that Prout's postulations were in part true, as discussed by Sir William Ramsey in 1897:

“…what we term the atomic weight of an element is a mean; that when we say the atomic weight of oxygen is 16, we merely state that the average atomic weight is 16; and it is not inconceivable that a certain number of O_2_ molecules have a weight somewhat higher than 32, while a certain number have a lower weight.”

(Quoted from Aston (1921)).

This thought hinted that the atomic weight of an element may not be conceived of a single weighted atom, and that the average atomic weight of the element consists of multiple versions (or isotopes) of the same compound with slightly varying atomic weights, which when taken together give the average which is measured. Despite this early recognition, physico‐chemical evidence of isotopes only first started to come to light in the early 20th century through the works of Sir Ernest Rutherford and Frederick Soddy studying the disintegration (or emanation, as it was often described) of radioactive elements (Rutherford & Soddy, 1902; Rutherford, 1903). Years of study of the radioactivity of these compounds highlighted a common finding throughout that there were series of radioelements that were chemically inseparable from each other, had similar chemical properties, yet differed in atomic weight. Soddy defined this within a communication in 1911 providing evidence of the “*perfect*” chemical similarities of two sets of elements; firstly that of mesothorium (AW as calculated by Soddy: 228.4), radium (226.4), and thorium X (224.2), while also that of thorium (232.4), ionium (230.5), and radiothorium (228.4) (Soddy, 1911), despite the elements within these sets having distinctly different masses. This was believed to be achieved by successive radioactive expulsions whereby the loss of a single alpha particle would shift the element two places lower in the periodic table and the further expulsion of two beta particles returns the element to its original position with the same intra‐atomic charge, but a different atomic weight to the original element (Soddy, 1913a). This led to the conception by Soddy that:

“…chemical homogeneity is no longer a guarantee that any supposed element is not a mixture of several different atomic weights or that any atomic weight is not merely a mean number.” (Fleck, 1957),

and following his informal discussions with Dr. Margaret Todd at a reception hosted by his Father‐in‐law Sir George Beilby, the term isotope was born to describe this phenomenon (Soddy, 1913a; Fleck, 1957; Nagel, 1982). Research over the following years provided further support for the presence of these isotopes. For example, it was found that a common product of some radioactive disintegration was lead. This lead, despite having identical chemical properties to common lead, which has an atomic weight of 207.15, radio‐lead produced from the disintegration of a variety of different sources (uranium, radium, thorium) was found to have a calculated atomic weight of between 206 and 210, suggesting the presence of different “radio‐isotopes” of lead (Soddy, 1913b; Soddy & Hyman, 1914). This “isotope” phenomenon was proven to not simply be due to samples of the elements tested being of unpure or differing origins, a very detailed investigation using samples of radioelements from different sources across the globe, showed that the lead produced from all these sources consistently had the same chemical properties as common lead, but a different atomic weight (Richards & Lembert, 1914). These important breakthroughs provided some of the first physical evidence of the existence of isotopes, a word now commonplace in the vocabulary of the scientific community.

**Figure 2:**
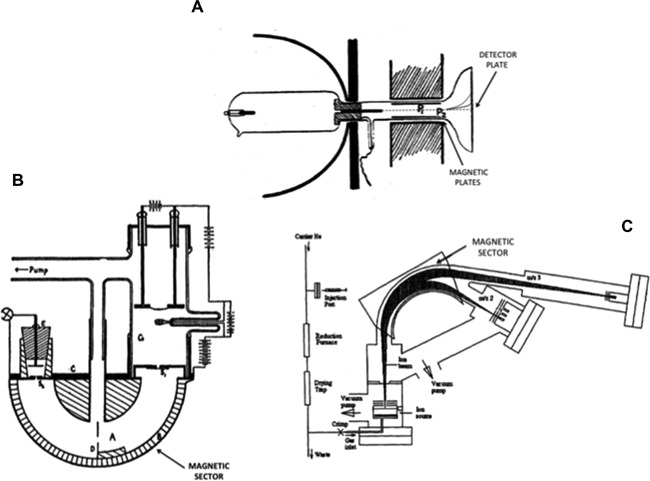
Evolution of the mass spectrometer over the years, (**A**) JJ Thomson's original mass spectrograph for analysis of positive rays (Reprinted with permission from Thomson, 1913 (copyright 1913, The Royal Society)), (**B**) Dempster's single magnetic sector instrument (Reprinted with permission from Dempster, 1918 (copyright 1918, American Physical Society)), (**C**) Present day set‐up for a commercial IRMS with magnetic sector, note the design differs very little from that of Dempster's original design (Reprinted with permission from Begley and Scrimgeour, 1996 (copyright 1996, John Wiley and Sons)).

**Figure 3:**
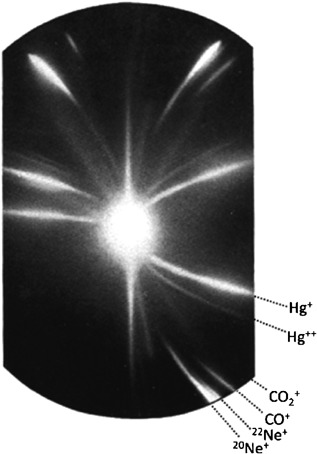
Photographic plate from JJ Thomson's original experiment into positive rays highlighting the presence of the Neon 22 isotope (Reprinted with permission from Maher, Jjunju, & Taylor, 2015 (copyright 2015, American Physical Society)).

From the pioneering work into radioisotope, it was, therefore, very eloquently suggested that if such a phenomenon is present among the heavy, unstable radioactive elements in the periodic table, surely the same should be possible among the smaller, *stable* elements (Soddy, 1913c). Work being performed around the same time as Soddy, investigating the properties of cathode rays was instrumental in supporting the existence of the radioisotopes stable cousin. It was observed that when small slits were placed in the cathode of a vacuum tube, rays of light from the gas contained within the vacuum tube could be seen streaming from the tube (Thomson, 1897; Goldstein, 1898; Wien, 1898). By placing powerful magnets along the path of these rays, they could be deflected, and through the measurement the amount of magnetic deflection obtained, the isotopic composition of the gas could be determined (Thomson, 1910, 1911, 1913). If one takes into account the simple design of this positive ray spectrograph instrument; electro‐magnets deflecting gases onto detector plates, it bears striking resemblance to today's modern isotope ratio mass spectrometers, and does not differ significantly to the magnetic sector instrument described by Dempster (1918) (see Fig. 2), highlighting that the basic design of the sector mass spectrometer has changed little since its initial inception. By placing a photographic plate in the path of the deflected positive rays, the isotopic composition of particles making up the rays could be determined as no two particles would strike the plate in same plane unless they had the same mass to charge ratio (or m/z; Thomson, 1913). This initial design was later refined by Francis W Aston (a former research assistant of Thomson's) and his colleagues at the Cavendish laboratory in Cambridge in order to improve the accuracy of the instrumentation, these developments are often attributed to what is now thought of as the first mass spectrograph (or mass spectrometer), the development, design, and implementation of which is described in detail by Aston in his Bakerian lecture of 1927 (Aston, 1927). During this time of initial design and development of the positive ray spectrograph, some intriguing results were revealed. Of the multitude of elements examined using this method, all except one would produce a single parabola detected on the photographic plate. Thomson, however, found in a sample containing neon that in addition to the line produced at mass 20, that corresponding to the known atomic weight of neon (Watson, 1910), there was an additional faint line at mass 22 (Thomson, 1907, 1913; see Fig. 3). Thomson believed that this line must be due to the gas of neon not being a simple gas, but a mixture of two different gases of atomic weights 20 and 22, with the greatest proportion being attributed to the gas of mass 20 (Thomson, 1913). While not being incorrect in his assumptions, the proportion of the masses 22 to 20 was always detected consistently even after prolonged purification of the gas samples (Soddy, 1913c), and it was soon attested through the work of Aston that this heavier mass was in fact an isotope of neon (Aston, 1919a, 1920) with Aston suggesting evidence of a potential third isotope of neon of mass 21 (Aston, 1921), this was confirmed much later with the actual composition of Neon gas being 90.4% ^20^Ne, 0.3% ^21^Ne, and 9.3% ^22^Ne (De et al., 2003). Within a few years, improved instrumental accuracy led to the discovery of a number of other naturally occurring stable isotopes (Aston, 1921), and as a result there are now up to 300 known stable and long‐lived isotopes (De et al., 2003).

While the discovery of the existence of these stable isotope species was no doubt a fundamental step forward, the successful isolation and incorporation of stable isotopes into different compounds for experimentation should be considered just as important. Much of this work was driven forward rapidly through the dedicated works of Messrs.’ Harold Urey and Rudolph Schoenheimer.

### A. The Birth of Stable Isotope Tracers

**Figure 4:**
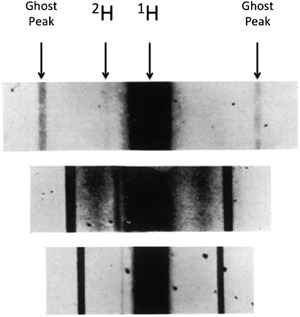
Harold Urey's evidence for the presence of the hydrogen isotope of mass 2, highlighted is the faint ^2^H line to the left side of the overexposed central ^1^H line with the symmetrical ghost peaks due to the overexposure at either side (Reprinted with permission from Urey et al., 1932b (copyright 1932, American Physical Society)).

Rudolph Schoenheimer trained as a physician in Berlin in 1922, before a keen interest in the physiology and pathophysiology of sterols drove him into the field of research (Guggenheim, 1991). His work was interrupted in 1933 by the Nazi party coming into power in his native Germany, leading to his subsequent emigration to the US and a post at Columbia University under Hans T Clarke (Guggenheim, 1991). This was an incredibly fortuitous appointment because currently installed at Columbia University was Harold Urey, a physicist who at the time was in the process of studying the separation and isolation of the stable isotopes (Urey, 1931; Murphy & Urey, 1932). Following work by Aston using mass spectrometry to determine the atomic weight of hydrogen (Aston, 1927), a discrepancy was observed with the calculation produced by Aston's work and that determined chemically (Urey, Brickwedde, & Murphy, 1932a). Chemically, hydrogen produced an atomic weight of 1.00777 ± 0.00002, while Aston's reduced to the chemical scale was 1.00756, outside the limits of technical error. Birge and Menzel (1931) discussed this discrepancy in the context of the recently identified oxygen isotopes, and suggested that this could only be due to the presence of a stable hydrogen isotope of low natural abundance (the abundance was predicted to be ∼1 part per 4500) which was as yet undetectable. Not long after this, Harold Urey was able to provide the first experimental evidence for the existence of this hydrogen isotope of mass 2 (Urey, Brickwedde, & Murphy, 1932a,1932b). Using different samples of hydrogen gas evaporated at different pressures and investigation of the predicted atomic spectra of ^2^H and ^3^H, faint lines were thus identified at the theoretical positions for ^2^H (no lines for ^3^H were seen). This was confirmed in the secondary gas samples tested to not be due to ghost peaks stemming from the saturated ^1^H band (see Fig. 4; Urey, Brickwedde, & Murphy, 1932b). Furthermore, upon calculation of the relative abundance of this ^2^H to the ^1^H line, a ratio of 1 part per 4,000 was obtained, interestingly very similar to that which was predicted by Birge and Menzel (Urey et al., 1932a). This discovery was paramount and efforts to utilize this isotope for biological purposes were keenly pursued by Urey with assistance from his former student David Rittenberg and Schoenheimer (Guggenheim, 1991). With the first series of papers published only 3 years later.

In order to achieve this, Schoenheimer and Rittenberg had to develop new methods for the preparation of the deuterium (as D_2_O; Rittenberg & Schoenheimer, 1935), introduction of the deuterium isotope into compounds of interest (Schoenheimer & Rittenberg, 1935a), as well as the development of methods required to analyze the deuterium content of this compound and associated metabolic products (Rittenberg & Schoenheimer, 1935). They initially noted that a rapid reaction takes place whereby if a compound is introduced into an atmosphere of deuterium in the presence of a catalyst (palladium), hydrogens on —OH, —NH2, —CHO, and alpha carbon positions, can readily exchange with deuterium, forming stably labeled compounds (Schoenheimer & Rittenberg, 1935a). Using these highly novel techniques, deuterated linseed oil was prepared and fed as part of a diet to mice, and Schoenheimer and Rittenberg were able to demonstrate that the ingested fat was utilized as an immediate source of energy with a small proportion going to fat depots as an energy store, which could then be utilized in times of need (Schoenheimer & Rittenberg, 1935b). This showed, for the first time, that fat metabolism was not a static inert process as was previously thought. The fat depots once formed were previously believed to be not usable, but in fact as highlighted by the work of Schoenheimer using stable isotope tracers, fat metabolism was a highly dynamic process.

Over subsequent years following these initial experiments, a great deal of information was obtained on the metabolism of fats and sterols using deuterium labeled compounds (Schoenheimer & Rittenberg, 1936a,1936b, 1937; Rittenberg & Schoenheimer, 1937a,1937b; Rittenberg, Schoenheimer, & Evans, 1937), furthermore successful deuteration of amino acids allowed for the additional probing of protein and amino acid metabolism (Foster, Rittenberg, & Schoenheimer, 1938b; Rittenberg et al., 1938). A total of 14 papers were produced under the general title of “Deuterium as an Indicator of Intermediary Metabolism” within only a few years using these deuterium labeled compounds (Rittenberg & Schoenheimer, 1935, 1937a,1937b; Schoenheimer & Rittenberg, 1935a,1935b,c, 1936a,1936b, 1937; Schoenheimer, Rittenberg, & Graff, 1935; Schoenheimer et al., 1936; Rittenberg, Schoenheimer, & Evans, 1937; Foster et al., 1938a; Rittenberg et al., 1938), this rapid and dedicated development highlighted the incredible utility of these “tracers.” Further progress was made with the successful isolation and concentration of the stable isotope of nitrogen (^15^N) by Urey in 1937 (Urey et al., 1937), this provided additional tools for investigating the metabolic fate of amino, amine, or amido‐containing compounds (Foster, Schoenheimer, & Rittenberg, 1939; Rittenberg, Schoenheimer, & Keston, 1939b; Schoenheimer & Rittenberg, 1939). By the end of the 1930s, the concentration, isolation, and introduction of the stable isotopes of hydrogen (^2^H; Urey et al., 1932a,1932b; Schoenheimer & Rittenberg, 1935a), carbon (Jenkins & Ornstein, 1932; Urey, Aten, & Keston, 1936a; Nier & Bardeen, 1941), nitrogen (Urey et al., 1937; Schoenheimer & Rittenberg, 1939), and oxygen (Urey, Pegram, & Huffman, 1936b; Cohn & Urey, 1938) into a range of compounds provided Schoenheimer and colleagues with an exquisite array of tools available for the probing of the biology of metabolism in health and disease. Through these techniques, a great deal of the knowledge regarding the understanding of mammalian metabolism was obtained, and much of what we understand today in terms of the control of metabolism derives from this early work. Among many of the processes that were identified during this intense period of development were the transport, interconversion, and deposition of fatty acids (Schoenheimer & Rittenberg, 1935b, 1936a; Barrett, Best, & Ridout, 1938; Cavanagh & Raper, 1939), the fate of dietary nitrogen (Foster, Schoenheimer, & Rittenberg, 1939; Rittenberg, Schoenheimer, & Keston, 1939b), the incorporation of dietary amino acids into tissue protein (Schoenheimer, Ratner, & Rittenberg, 1939), the (de)amination of amino acids (Schoenheimer, Ratner, & Rittenberg, 1939), cholesterol synthesis and metabolism (Rittenberg & Schoenheimer, 1937b), purine and pyrimidine metabolism (Plentl & Schoenheimer, 1944), formation of creatine and creatinine (Bloch & Schoenheimer, 1939), and the oxidation of fat stores (Schoenheimer & Rittenberg, 1936b). The findings provided during this intense period of development underpins the basis of our prevailing knowledge of mammalian metabolism today, helping to highlight the highly dynamic nature of metabolism and turnover of the body constituents (hence the title of Schoenheimer's posthumously published book; “*The Dynamic State of Body Constituents*” (Schoenheimer, 1942)), with substrate pools in constant metabolic flux.

Despite this rapid early development throughout the 1930s, progress was curtailed by two factors: firstly the onset of World War II, during which many scientists were involved in either fighting or projects to help the war effort, and secondly and rather tragically Rudolph Schoenheimer took his own life in 1941 following a battle with depression (Kennedy, 2001). Yet despite this, what Schoenheimer had managed to achieve in the preceding years remains extremely valid even today, and furthermore he left a legacy of dedicated, innovative scientists and collaborators who would continue where he left off. For example, Rittenberg continued investigations in protein synthesis, while also remaining heavily involved with all other aspects of metabolism (Rittenberg, Sproul, & Shemin, 1948; Sprinson & Rittenberg, 1949a, 1949b), with other protégés such as Konrad Bloch pushing forward with cholesterol and fat metabolism (Bloch & Rittenberg, 1942, 1943; Bloch, Berg, & Rittenberg, 1943; Bloch, Borek, & Rittenberg, 1946), and David Shemin continuing research into amino acid metabolism and heme biosynthesis (Shemin & Rittenberg, 1945, 1946; Shemin, London, & Rittenberg, 1948; London, Shemin, & Rittenberg, 1949). Unfortunately, following the conclusion of World War II, the use of stable isotopes declined, primarily due to the increased availability of radioisotopes (Rubin & Kamen, 1941), alongside the ease and sensitivity through which radioisotopes could be measured at the time (Kamen, 1947). However, there was also a limited availability of accurate analytical instrumentation for measuring stable isotopes. While deuterium‐containing compounds could be reduced to water and the deuterium content accurately measured by densitometry, refractive index (Zeiss interferometer), or the falling drop method (Rittenberg & Schoenheimer, 1935; Fenger‐Eriksen, Krogh, & Ussing, 1936; Cohn, 1995; Young & Ajami, 1999), isotopes like ^15^N nitrogen required extraction of the nitrogen as ammonia (Kjeldahl procedure), conversion to a gas, and introduction into a mass spectrometer for isotopic abundance (Rittenberg et al., 1939a; Schoenheimer & Rittenberg, 1939). These early mass spectrometers were not commonly available, and often required construction based on published information (Rittenberg et al., 1939a; Schoenheimer & Rittenberg, 1939). The war had led to the commercial availability of a number of long lived radioisotopes such as ^14^Carbon, which could be readily analyzed with high sensitivity and relative ease (Kamen, 1963) and this led to radio‐tracers superseding stable isotopes in subsequent years for the study of biological processes. However, this was not the end of the stable isotope tracer, future technological developments and safety concerns over the use of ionizing radiation in humans would soon bring stable isotopes back to the forefront of metabolic research.

## III. ANALYTICAL DEVELOPMENTS DRIVING METHODOLOGICAL PROGRESS

### A. Early Mass Spectrometry—From Physical Chemistry to Physiology

Following introduction of the first forms of mass spectrographs and mass spectrometers by Thomson (Thomson, 1907, 1910, 1911) and Aston (Aston, 1919b), there was an intense period of refinement and development over subsequent years. Many of these instruments at the time were limited to the identification and isolation of isotopes of different elements; therefore, improvements in resolution and detection were key areas of focus. The development of single focusing magnetic sector instruments by Dempster (Dempster, 1918) and further refinements in ionization in the form of electron ionization sources for gaseous material (Bleakney, 1929) led to advances such as the ability to use these instruments to study the complex molecular structures of hydrocarbons from as early as 1938 (Hustrulid, Kusch, & Tate, 1938). Resolution of these early single focusing instruments was still relatively poor. It was noted, however, that with the employment of both magnetic and electric fields within the instruments, termed double focusing (a technique which Aston had already been employing with his measurements), both directional and velocity focusing of the ions was possible, significantly improving the sensitivity and resolution of mass spectrometers (Mattauch & Herzog, 1934). Important refinements to the sector instrumentation by Alfred Nier during the 1940s and early 1950s (Nier, 1940, 1947) led to machines capable of considerably higher resolution than those developed prior to this; through a novel arrangement of the magnetic and electric sectors reducing interference in detection and ionization (Johnson & Nier, 1953). Using this machine and two compounds, CO_2_ and C_3_H_8_, both of mass 44, Nier was able to completely separate these compounds using his double focusing instrument (Nier, 1955). This demonstrated the powerful potential for application of mass spectrometry beyond that of the typical physical chemistry arena.

Despite these technical advances, many mass spectrometers remained the sole domain of physicists and physical chemists, where they were primarily focused on the understanding of the nature of atoms, elemental isotopes, and molecular ionization (Nier, 1989). Few outside these fields knew of these machines, or understood the potential application they could or would have (Nier, 1989; Griffiths, 2008). Furthermore, few mass spectrometers existed prior to 1940 and none were as yet commercially available. As transpired with many other scientific and engineering disciplines (such as the aeronautical and aerospace industries), the onset of World War II and in particular the Manhatten Project nuclear programme, had a substantial impact on the development, design, and construction of new and novel mass spectrometric equipment, with the first commercial machines being made available not long after the war through companies such as Metropolitan Vickers in the UK, Westinghouse and GE in the US, and Atlas Werke in Germany (Nier, 1989; Borman, 1998). It was around this time that the potential for mass spectrometry outside the disciplines of physics and chemistry was beginning to be truly realized. Alfred Nier, as had occurred to Harold Urey 10 years previous, realized that there was far more scope for mass spectrometers outside of physics. Using a technique engineered in Germany by Clusius and Dickel (Clusius & Dickel, 1938), Neir was able to produce ^13^C labeled methane at an enrichment of ∼10% (Bardeen, 1940; Nier & Bardeen, 1941). This ^13^C‐methane soon found its way into the hands of biologists and botanists who utilized it in the study of carbon metabolism of plants and bacterium (Wood et al., 1940). With one of the few mass spectrometers at the time sensitive enough to measures these carbon isotopes within his lab, Nier, alongside engineering new and novel mass spectrometers, found himself central to the early promotion of the multiple applications of mass spectrometric equipment as an analytical tool in other scientific disciplines, particularly in tandem with stable isotope tracers (Nier, 1989; Griffiths, 2008).

While the war drove great progress in mass spectrometry, it also drove forward greater understanding of the radioactive elements, in particular the discovery of the long‐lived radio‐isotopes such as carbon 14 (Kamen, 1963). Following years of dedicated work, it was shown that bombardment of a graphite probe by deuterons for 120h yielded a radioisotope of carbon with mass 14 (Ruben & Kamen, 1940; Rubin & Kamen, 1941), which upon further investigation possessed a very long half‐life (believed to be years or millennia initially at the time (Kamen, 1963), now known to be ∼5700 years). This discovery was incredibly important scientifically, particularly with regard to tracer technologies. Carbon is central to all biological processes; therefore, the study of its metabolism was key to understanding the biology of life itself. Hence, with its long half‐life, the ease with which it could be accurately measured using scintillation counters, ^14^Carbon, as with the discovery of ^2^H by Urey a decade earlier, was rapidly implemented into studies of metabolism (van Niel et al., 1942; Nahinsky et al., 1942; Barker & Kamen, 1945). This combined with the tritium radioisotope (Thompson, 1952) led to a significant decline in the use of their stable isotope cousins in the first 2 decades immediately following the war. Even stalwarts of the original stable isotope methods like Rittenberg saw the advantages of using these radioisotopes with long half lives and quickly took up these methods (Dische & Rittenberg, 1954). However, this was not the end for stable isotope tracers, there were aspects of metabolism where radioisotopes could not be used, such as nitrogen and oxygen metabolism. No long‐lived species of radioisotopes existed for these elements (the radioisotope ^13^N has a half life of 10minutes and ^15^O a half life of ∼120 sec; Audi et al., 2003); therefore, stable isotopes remained the only effective tool for studying metabolic processes involving N & O, for example, protein and amino acid metabolism, nitrogen balance, and liver function. ^15^N tracer studies, therefore, continued to be performed throughout the period of the radio tracer dominance (Wu & Bishop, 1959; Wu & Sendroy, 1959; Wu, Sendroy, & Bishop, 1959), yet it has been suggested that appreciation of the stable isotope tracer for metabolic measurement did not return until the late 1960s and early 1970s under the auspices of Waterlow, Garlick, and Millward (Young & Ajami, 1999). Now why did these tracers suddenly become prominent in research again? While not abundantly clear at the time, analytical devices had progressed rapidly since the work in Alfred Nier in the 1950s, indeed one may propose that this technological development alongside the realization of the potential health risks associated with radioisotopes may have driven this paradigm shift (Koletzko et al., 1997).

### B. Rapid Development and the Marriage of Separation

Up until the mid 1940s, mass spectrometers still maintained the same mass analyzer set‐up of sector instrumentation. While accuracy and resolution was ever increasing with new and novel designs for these instruments, they were limited. They were considered static analyzers, whereby the magnetic/electric fields were held constant meaning ions of different m/z would have different trajectories and hit the detector plate at different positions (Maher, Jjunju, & Taylor, 2015). In the late 1940s and early 1950s, great progress was made with the introduction of new types of mass analyzer, the time of flight or TOF analyzer in 1946 (Stephens, 1946) and the quadrupole in 1953 (Paul & Steinwedel, 1953). These analyzers were considered dynamic in nature, in which an electric field could be varied to allow the focusing of ions of different m/z into the same detector. TOF analyzers separate ions based on their velocities within an evacuated tube, the lower the m/z the quicker it arrived at the detector at the end of the flight tube, thereby m/z was represented as a function of time (Stephens, 1946). Little has changed from this initial design beyond minor modifications including reflectrons (Haberland et al., 1991; Cornett et al., 1992), and TOF analyzers are extensively used today, particularly in the fields of accurate mass, protein chemistry and proteomics/lipidomics (Tanaka et al., 1988; Fuchs, Süss, & Schiller, 2010). The quadrupole mass analyzer or mass filter in contrast uses strong electric fields to confine, isolate, and separate ions. First designed in 1953 by Wolfgang Paul and Helmut Steinwedel, the quadrupole mass analyzer (and QIT) were initially promoted as not requiring magnets for ion separation (however TOF does not require magnetic fields either; Paul & Steinwedel, 1953). The analyzer or filter consists of four rods arranged in opposing pairs with oscillating RF and continuous DC voltages applied across the rods with opposing charges for each (Mathieson & Harris, 1969). Ions entering the field of the quadrupole will have stable trajectory through the poles to the detector at a defined pair of RF and DC voltages, while those of other m/z would decay away, collide with the poles creating neutral fragments that are not detected (Dawson, 1986). Therefore, by varying the RF and DC voltages, different m/z will be obtained within a stable trajectory, making this type of mass analyzer very versatile, providing the ability to dynamically scan over a full mass range, while also allowing the fixing of RF and DC voltages for focusing on a selected m/z (Mathieson & Harris, 1969). The importance of QIT and quadrupole mass analyzers in modern analytical chemistry could form the basis of a single review; however, this is not the focus of this review, therefore interested readers are directed to the following dedicated review for more information (Dawson, 1976; March & Todd, 2015).

These new devices further enhanced the possible applications of mass spectrometry. However, it still remained the case that before introducing a sample into the mass spectrometer, particularly with complex matrices, a number of preparative steps were required to separate and purify the sample, with careful sample handling prior to introduction to the mass spectrometer (Sprinson & Rittenberg, 1949a). In 1952, James and Martin introduced the technique of gas‐liquid partition chromatography (GLC), using columns packed with a stationary phase of liquid silicone and stearic acid. With this, separation of complex mixtures of volatile acids and bases was possible (James & Martin, 1952a,1952b). It was not long before this separation technique was successfully coupled to a mass spectrometer, whereby compounds could be separated from each other in the GC and then introduced into the MS which acted as a detector (Abian, 1999). Much debate exists as to who was the first to combine these two techniques to form the first GC‐MS. In the mid to late 1950s, a number of investigators including Roland Gohlke and Fred McLafferty of Dow Chemicals successfully coupled a GC to a TOF MS providing rapid sensitive analysis of a mix of organic compounds (Gohlke, 1959), similarly Holmes and Morrell of Philip Morris, Inc. achieved the same coupling of a GC with a high resolution magnetic sector MS (Holmes & Morrell, 1957) and Beynon of Imperial Chemical Industries in the UK described the use of MS as a detector for GC in 1956 (Beynon, 1956). The importance of this relatively simple idea of coupling GC with MS analyzers cannot be underestimated to the physiologists and biologists who use these techniques today, particularly those utilizing stable isotope tracers. Many samples which require analysis for isotopic abundance in tracer work are contained within complex biological matrices consisting of numerous metabolites (such as tissues, blood, urine, cells etc…), therefore the ability to separate, isolate, and analyze these metabolites with relatively minimal sample work up by GC‐MS, made experiments easier to do, less time consuming, and cheaper to perform. Furthermore, electron ionization (EI) ion sources common to many mass spectrometers of the time provided fragmentation of the compounds upon ionization. In terms of stable isotope tracers, this allows greater understanding of metabolic pathways through positional isotopomer (isotopic isomers containing the same number of each elemental isotope but differing in position) analysis. By identifying the presence of a stable isotope tracer on a certain carbon position of a compound, for example, one can identify the pathways responsible for introducing this particular carbon into the molecule (Katanik et al., 2003). This was complimented by the additional introduction of “softer” ionization techniques like chemical ionization (CI) in the 1960s (Munson & Field, 1966), whereby a large amount of a reagent gas (ammonia or methane traditionally) which is ionized in the source reacts preferentially with the analyte of interest to create an ionized species of the analyte with minimal fragmentation or the formation of adduct ions with the reagent gas (Munson & Field, 1966), providing an alternative option for analysis by GC‐MS.

GC‐MS has become a very popular instrumentation set‐up, and is now commonplace within many analytical laboratories today. Many modern instruments utilize quadrupole mass analyzers (rather than TOF or magnetic sector), which, although suffering from limited mass ranges (50–1,000 m/z) and resolution (Dawson, 1986), permit the analysis of a wide range of compounds from relatively small amounts of sample (Medeiros & Simoneit, 2007). However, GC‐MS alone is only able to distinguish isotopic abundance ratios down to ∼0.1–0.5%, isotope ratio mass spectrometers on the other hand are capable of much higher accuracy for measuring discrete changes in isotopic abundance for carbon and oxygen isotopes to 0.005–0.02% (Table 2) (Matthews & Hayes, 1978). These instruments have changed little in design from the early instruments developed by Alfred Nier (McKinney et al., 1950); however, despite the improved levels of accuracy and precision for isotopic abundance measurement, in order to introduce a physiological/biological sample into the instrument, the compound still required extraction, purification, off line liberation of CO2, collection, and distillation prior to analysis by IRMS (von Unruh et al., 1974). This made the whole process expensive, time consuming, and prone to sample loss/contamination. Great progress, however, was afforded by Matthews and Hayes who, following on from the initial work by Tsuji (Tsuji, Masugi, & Kosai, 1975) and Sano a few years earlier (Sano et al., 1976), in 1978 successfully interfaced a gas chromatograph to a conventional sector mass spectrometer with a combustion interface, creating one of the first types of “isotope ratio monitoring gas chromatograph—mass spectrometers” (Matthews & Hayes, 1978). Using this technique, compounds were separated on a GC column, after which the effluent was directed online to a 750°C cupric‐oxide combustion furnace, whereby the compounds were combusted to N_2_ (following reduction from nitric oxide via a copper filled reactor positioned directly after the main combustion furnace) and CO_2_ gases before being directed to the MS for ^15^N/^14^N or ^13^C/^12^C ratio analysis (Matthews & Hayes, 1978). Further work in the development of these machines continued during the early 1980s in the UK, with Preston & Owens (1983) developing continuous flow‐IRMS instrumentation, describing the interfacing of an automatic nitrogen analyzer to an isotope ratio mass spectrometer, with the first description of the modern day GC‐C‐IRMS instrumentation configuration by Barrie at VG instruments in 1984 (Barrie, Bricout, & Koziet, 1984). It was not long until such instrumentation became commercially available in 1988 (Brand, 1996). This was a significant step forward in terms of stable isotope analysis, physiologists, and biologists now, approximately a quarter of a century after introduction to the field, started to have access to the necessary tools for the next chapter in stable isotope tracers to begin. With two complimentary sets of instrumentation (and the introduction of microcomputer control and processing; Schoeller & Klein, 1979) in GC‐MS and GC‐IRMS, isotopic abundance could now be measured readily in complex biological matrices from as low as 0.005 to greater than 90 APE. Of note, however, with modern instrumentation the LOD difference between GC‐MS and GC‐IRMS has become narrower, with many GC‐MS instruments (especially GC‐MS/MS see Table 2) able to detect very low levels of enrichment which in the past would have only been possible with IRMS, particularly in combination with multiply labeled tracers such as ^13^C_6_ or D_8_ phenylalanine (Bornø, Hulston, & van Hall, 2014b; Hines et al., 2015).

**Table 2:**
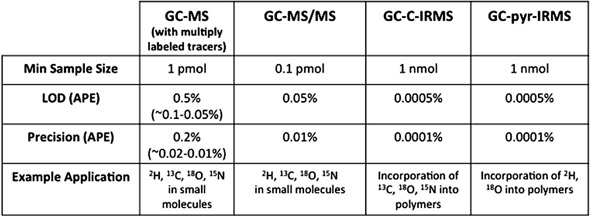
Comparison of present day gas chromatography based mass spectrometry equipment commonly used for stable isotope tracer analysis

Stable isotope tracers over subsequent years helped to define substrate kinetics within a number of areas of metabolism from lipids to carbohydrates to vitamins and minerals. However, an area where these techniques have probably produced the most impact has been amino acid‐protein metabolism. Therefore, much of the remainder of this review will focus on this area in particular. For more details on these other areas, the reader is guided to the following reviews (Turnlund, 1991; Kalhan, 1996; Bluck, 2009; Ecker & Liebisch, 2014).

### C. Methodological Development—The Abundance of Stable Isotope Tracer Techniques for Protein Metabolism Increases

**Figure 5:**
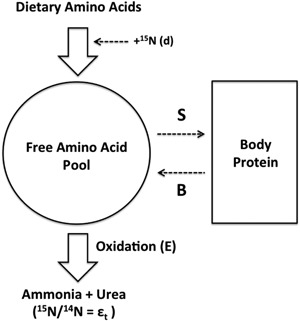
Diagrammatic representation of the two pool model for calculating protein turnover using the end product method as devised by Waterlow (adapted from Duggleby & Waterlow, 2005).

With techniques in place, and analytical equipment capabilities for measurement of isotopic abundance in a wide variety of sample matrices and detection ranges, stable isotope tracers underwent a rapid renaissance throughout the 1970s and 1980s. Work by John Waterlow's group at the MRC's Tropical Metabolism Research Unit was one of the primary early drivers involved in this new chapter for stable isotope tracers (Millward & Stephen, 2011). With a keen interest in the influence of protein deficiency and malnutrition on organ function, particularly in infants and young children, this group developed a number of stable isotope approaches to study protein metabolism. In 1969, Picou, Taylor‐Roberts and colleagues introduced an approach whereby ^15^N Glycine was provided either orally in repeat doses (Picou & Taylor‐Roberts, 1969) or via continuous intravenous (iv) or intragastric (ig) infusion (Picou, Taylor‐Roberts, & Waterlow, 1969) to provide a measure of whole body protein turnover. The continuous administration over ∼30 hr created an eventual isotopic steady state, from which the rate of protein turnover could be calculated from the ^15^N abundance in the excreted “end product;” urinary urea (Picou, Taylor‐ Roberts, & Waterlow, 1969). While this technique was not essentially new, Rittenberg had described similar end product techniques in the late 1940s (Sprinson & Rittenberg, 1949a; San Pietro & Rittenberg, 1953a, 1953), these previous methods required complex mathematical analyses alongside a long list of assumptions. Waterlow took it upon himself to devise the above stochastic methods which involve minimal assumptions and modeling to describe protein metabolism, with the major assumption of the technique being that enrichment of the end product (i.e., urea and ammonia) reflects that of the amino acid pool taken up into the body proteins (Fig. 5), and from equations (1) and (2) below, protein turnover can be calculated based on these assumptions:

(1) $$S=Q-E_{t}$$

(2) $$Q=d/\epsilon_{t}$$

(where Q = flux, S = synthesis, d = dose of tracer, ϵ_t_ = ^15^N enrichment in the total collection, and E_t_ is the total N excreted).

These methods, since this original work have been extensively validated, adapted, and improved upon on numerous occasions and have stood the test of time and continue to be applied today (Duggleby & Waterlow, 2005).

The benefit of such whole body protein turnover measurement is the ease of isotopic administration and minimally invasive sampling procedures employed (i.e., only urine collection). This made the method ideally suited to the work of Waterlow and colleagues in the 1970s, who were investigating malnutrition and associated disease in infants (Millward & Stephen, 2011). Yet, whole body measurements are limited, reflecting the turnover in all the tissues of the body, unable to distinguish from which protein pools/tissues any measurable differences are originating from. In order to do this, samples are needed directly from the protein pools/tissues of interest, to determine the amount of stable isotope tracer that has been incorporated/lost within this protein pool. These types of methods are based on the precursor‐product approach originally proposed by Zilversmit (1960), here a continuous infusion of an amino acid (or other appropriate substrate) tracer is infused until the amino acid pool reaches a steady state plateau, after which tissue samples are obtained and the level of tracer present in the bound protein provides a measure of the rate of incorporation or synthesis if the level within the precursor pool (or appropriate surrogate) is known (as described at the beginning of this review). Waterlow & Stephen (1967, 1968) developed this method using [U‐^14^C]‐Lysine, and were successfully able to apply it to measure specific rates of synthesis of plasma, liver and muscle proteins in rats. Further refinements of this technique were provided a few years later by Peter Garlick (1969), highlighting the powerful potential for these techniques in the measurement of muscle protein synthesis in particular, a tissue which contributes significantly to the control of overall metabolic health (Garlick, 1969). The development of GC‐IRMS instrumentation as described earlier was key to the widespread application of the precursor‐product approach, permitting use of stable rather than radio‐labeled tracers. Tissue protein, such as muscle, turnover at a relatively slow rate, therefore the amount of tracer incorporated into this protein will be minimal even over a period as long as 24 hr; hence, sensitive methods for measuring absolute changes in isotopic abundance over time are essential for accurate measurement. Dave Halliday was one of the first to attempt these precursor‐product tracer methods using L‐[α‐^15^N]‐Lysine infused over 30 hr, collecting serial muscle biopsy samples to determine rates of muscle protein synthesis (Halliday & McKeran, 1975), with rates reported at between 1 and 2%/d, indistinguishable from the rates reported routinely today with more sophisticated MS instrumentation.

These initial techniques required continuous 30 hr infusions to be performed, creating a very lengthy experimental set‐up, due to the time it took for the amino acid tracer pool to reach plateau, reported by Halliday as ∼12–14 hr (Halliday & McKeran, 1975). This infusion could be shortened somewhat by the inclusion of a bolus prime of tracer prior to the beginning of the constant infusion phase. Rennie et al. (1982) reported that using such an approach, plateau enrichment could be reached within ∼2 hr, significantly shortening the study protocol length (Rennie et al., 1982). There are a number of requirements for the measurement of muscle protein synthesis using the precursor‐product approach in that the level of enrichment in both the bound protein amino acid pool; the product, and the level of enrichment of the related precursor to this product must be measured for calculation of the FSR. The true precursor of muscle protein is the aminoacyl‐tRNA; however, the aminoacyl‐tRNA pool is very small, labile, and difficult to access particularly in humans, therefore a surrogate precursor that reflects the enrichment in the aminoacyl‐tRNA pool needed to be identified. In a couple of elegant studies, when using leucine stable isotope tracers either venous plasma KIC (the oxidation product of leucine) or the free intracellular leucine enrichments were found to serve as robust surrogates for the leucyl‐tRNA (Watt et al., 1991), with free intracellular phenylalanine enrichment acting as an suitable surrogate for phenylalanyl‐tRNA when using phenylalanine stable isotope tracers (Baumann et al., 1994). In a bid to overcome the issues with precursor measurements, “flooding dose” techniques were developed where a large amount of labeled amino acid is given together with the unlabeled amino acid as a single bolus, this technique should in theory result in rapid equilibration of both the intracellular and extracellular amino acid pools to similar enrichments, inclusive of the pool used for charging the tRNA, and therefore enrichment of plasma free amino acids should accurately reflect that of the tRNA pool also (Garlick et al., 1989). However, this technique also has limitations, it was demonstrated that providing a large bolus of amino acids (and essential amino acids specifically) may result in stimulation of muscle protein synthesis thereby providing erroneously high rates (Smith et al., 1992, 1998). As such this led to great debate as to the optimal method for measuring MPS with stable isotope tracers in the 1990s (Garlick et al., 1994; Rennie, Smith, & Watt, 1994); however, the primed‐constant infusion method is still considered the gold standard despite recent publications arguing to the contrary (Caso et al., 2006) and alterations to the flooding dose methods in an aid to improve the method (Tuvdendorj et al., 2014). With further development in subsequent years through the increasing sensitivity of both GC‐MS and GC‐C‐IRMS systems and the introduction of multiply labeled amino acids tracers (Patterson et al., 1991; Patterson, Zhao, & Klein, 1998; Patterson & Wolfe, 1993), it is now possible to perform stable isotope tracer measurement of muscle protein synthesis accurately over measurement periods as short as 45–60 min (Atherton et al., 2010; Atherton & Smith, 2012). Indeed, the use of multiply labeled tracers has also provided the opportunity for groups to avoid investing in expensive IRMS equipment in order to run isotope tracer work in humans, as current GC/LC‐MS instrumentation can provide adequate measurement sensitivity under certain conditions (Patterson et al., 1991; Zabielski et al., 2013). These stable isotope tracer techniques have proved vital in uncovering the temporal nature of the response of muscle to feeding, underscoring the primary importance of amino acids in nutrition. It was initially shown that following a mixed meal, MPS was significantly increased in healthy young individuals (Rennie et al., 1982; Halliday et al., 1988), and it was soon discovered that this stimulation was primarily driven through the amino acid content of these meals (Bennet et al., 1989b), with further refinement providing specific evidence for the key role of EAA (Smith et al., 1992, 1998), in particular the BCAAs, with leucine providing the most potent stimulatory properties (Wilkinson et al., 2013; Bukhari et al., 2015). The further introduction of two, three, and four pools A–V tracer kinetics by Wolfe and colleagues throughout the 1980s and 1990s for measuring synthesis and breakdown across tissues (Barrett et al., 1987; Gelfand & Barrett, 1987; Biolo et al., 1994, 1992b; Miller et al., 2004) alongside development of the fractional breakdown rate measurements based on tracer decay kinetics from the protein pool (Zhang et al., 1996) provides a full arsenal of stable isotope tracer techniques to investigate the regulation of protein turnover (i.e., synthesis and breakdown by nutrition, exercise, and hormones in health and disease) throughout the lifespan. Thus, these techniques have provided a vast array of data on metabolism since their introduction and are becoming increasingly common analytical tools. By the end of the 20th century, physiologists/biologists had the ability to intricately investigate many aspects of metabolism in a wide array of metabolic pathways, often through the addition of a single stable isotope tracer but more commonly utilizing multiple tracers to understand the relative importance of particular pathways.

### D. Liquid Chromatography—It Took a Long Time, But It Was Not Forgotten

There has been very little discussion relating to the use of liquid chromatography (LC) as a powerful separation technique in this review up until now. Despite first being described almost half a century prior to GC (Tswett, 1903), it took another 40 years before it became a useful separation tool following development by Martin and Synge (one part of the group who went on to develop GC 10 years later; Martin & Synge, 1941), and a further 30 years before LC was successfully coupled with a mass spectrometer (Lovins et al., 1973). By this time, GC‐MS had long been established and was already a widespread technique within laboratories. Despite this, LC has a number of benefits over GC. For example, GC separation generally requires chemical transformation/derivatization of thermolabile/polar compounds into thermally stable compounds which will enter the gas phase (Godin, Fay, & Hopfgartner, 2007). This can introduce contaminants in sample preparation phases, and requires the understanding that the compound being detected is no longer the same as the original compound, but is in a different chemical form (Meier‐Augenstein, 1999a,1999b; Shinebarger, Haisch, & Matthews, 2002). Furthermore, thermal degradation can also occur to some compounds within the heated elements of the GC if not careful, while not all compounds are capable of being analyzed via GC‐MS, even following chemical modification and the m/z range is limited with GC, that is, to ∼1,000 amu (Buser et al., 2000). Unlike GC, LC does not require samples to be volatile to be analyzed, and in most cases can be analyzed following simple liquid/liquid extraction and injection without the need for prior derivatization (Zheng, Jiang, & Zeng, 2014), although in some cases derivatization may prove beneficial (Nakamura et al., 2015). Yet, one of the major reasons for the delay in coupling LC to MS was the issue of how to introduce the sample into the MS as it was contained within a large volume of liquid solvents flowing at very high rates, making it incompatible with high vacuum mass analyzers (Abian, 1999). Initial attempts used either deposition of solvent on a solid probe, after which the solvent was removed via evaporation prior to introduction to the MS (Lovins et al., 1973), or the moving wire/belt interfaces, which following deposition of the solvent on the moving wire, it was evaporated through a heating mechanism and the sample transported to the MS where it was desorbed and analyzed (Scott, Munroe, & Hess, 1974; McFadden & Schwartz, 1976). Over subsequent years, a number of different interfaces were developed from direct liquid introduction (Sugnaux, Skrabalak, & Henion, 1983), to fast atom bombardment (Caprioli, Fan, & Cottrell, 1986) to thermospray ionization (Blakley, McAdams, & Vestal, 1978), but it was not until the introduction and commercialization of atmospheric ionization in the form of atmospheric pressure chemical ionization (APCI; Horning et al., 1973) and electrospray ionization (ESI; Yamashita & Fenn, 1984) that LC‐MS became a practical, useful, and mainstream application for use with stable isotope tracers (van Eijk et al., 1999; Meesters, Wolfe, & Deutz, 2009). These ionization techniques benefit from being performed under atmospheric pressure (rather than high vacuum as in EI), providing the ability to produce intact ions of polar, non‐ volatile, thermolabile compounds that may not be applicable to classical ionization techniques such as EI (Abian, 1999). These techniques have become increasingly important in areas of analysis involving large biomolecules such as lipids, peptides, and proteins (Fenn et al., 1989); however, it should be noted that there are notable matrix effects associated with these LC‐MS ionization techniques, which can lead to significant ion suppression and interference with sample analysis (Trufelli et al., 2011). Something that is less common with GC‐MS and EI‐based ionization. The number of uses and publications of LC‐MS and stable isotope tracer techniques is steadily growing, with studies highlighting the now comparable sensitivity of LC‐MS techniques with the more established GC‐MS and GC‐IRMS techniques for measurement of substrate tracer kinetics and turnover (Godin et al., 2008; Zabielski et al., 2013; Bornø & van Hall, 2014; Bornø, Foged, & van Hall, 2014a; Bornø, Hulston, & van Hall, 2014b; Hines et al., 2015). This high level of sensitivity and selctivity of modern LC‐MS instrumentation has, in part, been greatly influenced by the introduction of tandem MS or MS^n^ techniques which allow the performance of selective reaction monitoring analysis or SRM (Colangelo et al., 2013). SRM is typically performed using triple quadrupole MS instruments (however can also be performed using other tandem MS configurations such as QTOF), whereby three quadrupole are aligned in series prior to the detector. Following ionisation of the compound in the ion source, the first quadrupole acts as a mass filter allowing passage of a predefined precursor ion to the second quadrupole which is in fact a collision cell containing an inert gas (e.g., argon), where the precursor ion is further fragmented before entering the final quadrupole which acts as another mass filter to select a predefined fragment ion (Levesen & Schulten, 1976; Kondrat & Cooks, 1978). This high level of selectivity provided by this technique significantly increases the signal to noise ratio by removing any background or interfering signals from the sample, thereby increasing the sensitivity of the equipment when analysing compounds within complex matrices such as biological samples (Kondrat & Cooks, 1978; Addona et al., 2009). Modern LC‐MS machines can further increase this selectivity by performing a series of SRM sequentially, a process called multiple reaction monitoring or MRM (Addona et al., 2009). Moreover, with the introduction of benchtop high resolution LC‐MS (Eliuk & Makarov, 2015) and the coupling of LC to combustion‐IRMS (Godin et al., 2005; Godin, Fay, & Hopfgartner, 2007; Godin et al., 2008), LC‐MS is now on par with GC‐MS techniques for use with stable isotope tracers, with sales of LC‐MS exceeding those of GC for the first time and likely to continue that way for years to come. Tools for isotopic analysis are now at a premium, and the potential for analysis of compounds seemingly knows no limits.

## IV. FULL CIRCLE—PRESENT DAY USES AND APPLICATIONS OF STABLE ISOTOPE TRACERS FOR STUDY OF *IN VIVO* PROTEIN METABOLISM

Stable isotope tracers are now at the forefront of the majority of physiological/biological mechanistic studies performed *in vivo*, and are complimented by the wealth of methodologies and techniques for determining flux through metabolic pathways and rates of substrate/pool turnover that are available, alongside the numerous analytical platforms which are now commercially available for the measurement of isotopic abundance. As a result, it is now possible to use stable isotope tracers for as diverse applications as medicine (Chan, Barrett, & Watts, 2004), diagnostics (Bonfrate et al., 2015), ecology (Garcia‐Anton et al., 2014), geology (Menicucci, Matthews, & Spero, 2013), forensics (Meier‐Augenstein et al., 2011), and art (Fortunato, Ritter, & Fabian, 2005), among many others. It is doubtful that even Schoenheimer himself could have envisaged where his experiments with deuterium in the 1930s would have led to today. While there are now a vast array of substrate‐specific stable isotope tracers available, from numerous commercial vendors, for probing multiple metabolic pathways and processes, it seems in terms of assessing metabolic function that the approaches have come full circle since the days of Schoenheimer and Rittenberg. Over the past few decades, there has been great interest in the potential of one of the first stable isotope tracers to be introduced; deuterium oxide (D_2_O), and its application has undergone somewhat of a renaissance.

The use of D_2_O as a stable isotope tracer allowed the first insights into the dynamic nature of substrate utilization and flux *in vivo* (Schoenheimer & Rittenberg, 1936b). While this tracer was intermittently utilized over subsequent years in either its stable or radiolabeled (tritium oxide) forms (Krogh & Ussing, 1937; Stekol & Hamill, 1937; Ussing, 1937, 1938, 1941; Jungas, 1968; Humphrey & Davies, 1975, 1976). The benefits of D_2_O as a stable isotope tracer only really began to truly be recognized in the last decades of the 20th century. Building upon the knowledge generated by researchers utilizing heavy water for measurement of body water pool size (Hevesy & Hofer, 1934), energy expenditure (Lifson, Gordon, & McClintock, 1955; Schoeller & van Santen, 1982) and aspects of plant protein metabolism (Humphrey & Davies, 1975, 1976), it was soon realized, through the dedicated work of Marc Hellerstein and Stephen Previs (working independently of each other), that D_2_O could overcome a number of the issues related to the use of traditional substrate‐specific stable isotope tracers. Administered either orally (Previs et al., 2004; Gasier, Fluckey, & Previs, 2010; Robinson et al., 2011; Gasier et al., 2012; MacDonald et al., 2013; Wilkinson et al., 2015b, 2014; Decaris et al., 2015) or intravenously (Emmanuel et al., 2011), continuously (Robinson et al., 2011; Brook et al., 2015; Decaris et al., 2015) or as a single bolus (MacDonald et al., 2013; Wilkinson et al., 2015b, 2014), D_2_O rapidly equilibrates with body water (within ∼20 min in rodents; Dufner et al., 2005 and up to 1–2 hr in adults humans (IAEA Human Health Series, 2011)), this creates a homogenous, slowly turning over, precursor pool available for use by multiple substrates. The deuterium from the body water can then be incorporated onto different substrates at stable C—H positions through biological reductions during *de novo* synthesis, and the metabolic flux of these substrate pools can be calculated from the measurement of the amount of the label that is incorporated (Hellerstein, 2004). Unlike the substrate‐specific tracer techniques traditionally used, D_2_O provides the advantage of measuring the turnover of multiple pools simultaneously with the sole administration of a single stable isotope tracer (Hellerstein, 2004). Furthermore, the homogenous distribution among the body water pool provides an easily accessible and measurable surrogate precursor pool (Dufner & Previs, 2003), which due to the relatively slow turnover of the body water pool, allows for the measurement of metabolic turnover over periods of hours–days–weeks–months (Robinson et al., 2011; Gasier et al., 2012; Wilkinson et al., 2015b, 2014; Brook et al., 2015).

Initial development of these D_2_O tracer methods in the 1980s and 1990s concentrated primarily on utilizing them for the measurement of glucose and lipid metabolism. When introduced *in vivo*, deuterium from D_2_O can be incorporated onto a number of different carbon positions dependent on which metabolic pathway is contributing to glucose production. For example, work by Chandramouli et al. (1997) determined that deuterium labeling at carbon position 5 in glucose was solely due to gluconeogenesis, while that of carbon position 2 was the result of both gluconeogenesis and glycogenolysis (Chandramouli et al., 1997), allowing relative contributions of each pathway to glucose production to be determined simply by providing D_2_O and measuring positional deuterium isotopomer labeling, and the ratios between the deuterium labeling found at carbons 5 and 2 (Previs & Brunengraber, 1998). Alongside this methodological development in glucose metabolism, there was growing interest in the use of D_2_O for measuring lipid turnover, one of the areas in which D_2_O was initially utilized by Schoenheimer (Schoenheimer & Rittenberg, 1936b). By measuring either or both of the deuterium labeling, following provision of D_2_O, in lipid species bound fatty acids (Diraison, Pachiaudi, & Beylot, 1997; Strawford et al., 2004) and triglyceride glycerol moieties (Turner et al., 2003, 2007), rates of *de novo* lipogenesis and triglyceride synthesis can be easily determined.

During these intense periods of method development, genesis of mass spectrometry equipment that would become vital for this tracer technique to flourish continued. While the flux of glucose and fatty acids is relatively rapid, there was growing interest in the implementation of D_2_O tracer techniques in slower turning over pools such as skeletal muscle protein and DNA. Critical to this is having instrumentation with sufficient sensitivity and accuracy for the assessment of small shifts in isotopic abundance. The majority of amino acids can be labeled at their alpha carbon positions following provision of D_2_O (Herath et al., 2011), this means that by isolating the protein pool of interest and measuring the amount of deuterium label at the alpha carbon position in the protein bound amino acids, a rate of protein turnover can be determined (Dufner & Previs, 2003; Gasier, Fluckey, & Previs, 2010). This amount of labeling is likely to be low, however, due to the slow rate of turnover of most tissue protein pools, and highly dependent on the amount of D_2_O provided to enrich the body water pool (for humans generally restricted to <2%, with this distributed evenly across multiple substrate pools not only that of protein), and the time period over which the measurement is taking place, that is, the longer the period, the greater the bound amino acid protein labeling. However, a number of techniques have been developed whereby the amount of bound enrichment being measured can be somewhat magnified, simply by choosing the appropriate amino acid to monitor. The non‐essential amino acids alanine, glycine, glutamate, and glutamine, unlike other amino acids, can be labeled by deuterium from D_2_O on multiple carbon positions (Busch et al., 2006; Herath et al., 2011); for example, up to four in total in alanine, one at the alpha carbon position; as with all other amino acids, and three at the beta carbon position, thereby providing magnification of up to fourfold for bound deuterium enrichment compared to other amino acids (Busch et al., 2006). Therefore, by using alanine as the product amino acid, and by ensuring the tissue protein pool being measured is turning over at a slower rate than the intracellular pool of alanine (as occurs with skeletal muscle protein), standard GC‐MS is sufficiently sensitive to use for analysis and numerous published methods have highlighted this approach (Gasier et al., 2012, 2011; Robinson et al., 2011; Miller et al., 2013). While this is practical for extended periods of measurements (weeks/months; with the period of measurement only restricted by the rate of turnover of the tissue pool being measured) or with high oral D_2_O administration (up to 2% of body water in humans; Robinson et al., 2011), the lower levels of tracer incorporation encountered with shorter periods of measurement (hours–days) or in cases where oral dosing of D_2_O is quite low (it is important to note that there are a number of side effects associated with consumption of D_2_O such as nausea, vertigo and light headedness, and therefore in most cases small doses over prolonged periods are preferred; Jones & Leatherdale, 1991) there will be an increased level of analytical error and variability of turnover rates (Fluckey et al., 2015; Wilkinson et al., 2015a). In contrast, IRMS provides the level of analytical sensitivity and dynamic range to facilitate these sensitive measurements. However, despite commercial GC‐C‐IRMS instruments being available from the late 1980s, difficulties with online combustion and interference of helium carrier gas with the H_2_ ion detection made high precision measurement of delta H_2_ by IRMS difficult, and it was not until the work of Tobias et al. (1995), and Begley & Scrimgeour (1996, 1997) that these issues were resolved. Tobias initially used a sequential in line set up of two reactor ovens to isolate H_2_ gas. The initial reactor involved conventional combustion with an oven held at 850°C with copper oxide (CuO) and Platinum (Pt) metal which converted the organic compounds to CO_2_ and H_2_O, this was then directed into a second oven containing a nickel catalyst at 850°C for reduction of water to H_2_ gas (Tobias et al., 1995). This gas was then passed through palladium filters to aid separation of the H_2_ gas from the He carrier in the ion source (Tobias et al., 1995). Following on from this initial work, Begley & Scrimgeour (1997) developed the online combustion further using a single reactor set‐up of an alumina nickel wired tube heated to 1,050°C (pre‐coated with hexane carbon) to directly pyrolyse compounds to CO and H_2_, this was based on previous work by the same group showing successful pyrolysis of water to CO and H_2_ using nickeled carbon (Begley & Scrimgeour, 1996). At temperatures of 1,050°C or above, this method was found to be 95% efficient (Begley & Scrimgeour, 1997). Post‐pyrolysis chromatographic separation and water trapping, alongside a novel design continuous flow IRMS, allowed good separation and high precision measurement of H_2_ with He as the carrier gas (Begley & Scrimgeour, 1997). These advances in online GC‐Pyrolysis‐IRMS instrumentation provided impetus for the widespread use of deuterated stable isotope tracers including D_2_O; these systems are now commercially available for use with most modern GC‐IRMS instrumentation. As such methods have been developed using only small oral doses of D_2_O (∼150 g, which provides a low level of isotopic enrichment in the body water pool), rates of muscle protein synthesis can be accurately measured using this pyrolysis‐IRMS instrumentation over 2–10 days (MacDonald et al., 2013; Wilkinson et al., 2014), in response to anabolic stimuli (e.g., unilateral resistance exercise; Wilkinson et al., 2014), and with appropriate dosing (∼400 g; consumed in multiple doses prior to measurement period to avoid nauseous effects) the approach is sensitive enough to measure MPS over periods as short as 3 hr, showing comparable rates to those obtained using primed continuous iv amino acid tracer techniques (Wilkinson et al., 2015b). As such, these validity studies have highlighted this wide applicability, sensitivity, and robustness of the D_2_O technique providing an important new tool for biological research that allows its implementation within populations where other traditional amino acid techniques may be contra‐indicated or difficult to apply (e.g., adolescence, frail elderly, ICU patients). Further to this, work undertaken by Marc Hellerstein and colleagues also highlighted the utility of D_2_O combined with IRMS for quantifying DNA turnover rates following deuterium labeling of deoxyribose moieties (Busch et al., 2007; Voogt et al., 2007). D_2_O is a truly multivalent stable isotope tracer (capable of tracing protein, glucose, lipid, nucleotide metabolism), that with careful consideration of kinetics (turnover rates of the pool to be measured) and modeling parameters alongside identification of true precursor labeling (through an easily accessible surrogate measure), can act as a universal cost‐effective tracer for monitoring multiple metabolic pools simultaneously, providing unique and detailed insight into how systems are effected/perform under a number of physiological/disease conditions. Moreover, this tracer has been shown to be able to provide information on protein breakdown rates also, a historically very difficult aspect of metabolism to quantify accurately (Holm et al., 2013). While clearly a highly advantageous tracer methodology, D_2_O like all other tracer techniques relies on a number of assumption and modeling caveats and therefore is not by any means the perfect measure. Details regarding these in more details can be found in the following publications (Previs & Kelley, 2015; Zhou et al., 2015a).

### A. The Age of “OMICS” and MS Arrives

While stable isotopes have helped drive some of the initial developments in MS technologies, some of the more recent developments have been in part due to the increasing interest in systems biology and associated OMICS technologies, whereby a holistic view of the molecules and interaction of these molecules and systems within the cell, tissue or organism as a whole can be described (Horgan & Kenny, 2011). Generally, OMICs can be organized into a top down pathway, beginning with genomics, the study of the protein encoding genes and the mRNA regulating the translation of these proteins (transcriptomics). Leading then to proteomics, the study of the proteins being expressed through the up/down‐regulation of associated genes, which will provide more valuable information than genomics alone, as a single gene can lead to the translation of many different proteins. Finally, there is metabolomics, the study of global metabolite profiles within the organism, this is the final product of the OMICS pathway and can provide powerful insight into phenotypic changes relating to the organism or tissue, and when combined with other OMIC platforms can provide a vast amount of information about health and disease (Claudino et al., 2007; Horgan et al., 2009; Valdes, Glass, & Spector, 2013). Indeed, the expansion of both the fields of proteomics and metabolomics has bolstered the development and innovation in MS technologies in recent years, through the need to accurately identify, isolate, and separate the vast array of proteins and metabolites from complex matrices efficiently and with high resolution (Cravatt, Simon, & Yates, 2007; Bachi & Bonaldi, 2008; Dunn et al., 2011).

LC‐MS equipment development contributed significantly to the proteomic era particularly through the development of soft ionization techniques such as ESI and matrix‐assisted laser desorption ionization (MALDI). These techniques allowed the ionization of fragile high molecular weight polar molecules, such as proteins, up to molecular weights of 100,000 Da or greater (Karas & Hillenkamp, 1988; Tanaka et al., 1988; Fenn et al., 1989), and when combined with the developments in new MS configurations and mass analyzers, for example, quadrupole‐TOF (Q‐TOF; Hopfgartner et al., 1999; Chernushevich, Loboda, & Thomson, 2001), quadrupole‐ion trap (QIT; Jonscher & Yates, 1997), triple quadrupole (QQQ; Kuksis & Myher, 1995), and TOF‐TOF (Cotter, Griffith, & Jelinek, 2007) configurations, performance and resolution of these machines has been greatly improved (Yates, 2004). In addition, the ability to induce fragmentation of their constituent peptides greatly assisted in identifying sites of modification of the protein, such as acetylation (Papac & Shahrokh, 2001). Improved protein–peptide sequencing, in addition to increasing resolution and separation in the form of micro (Emmett & Caprioli, 1994) and nanoflow LC (Shen et al., 2004, 2002), as well as the more recent development of benchtop Fourier transform (FT) MS instruments, such as the orbitrap (Makarov, 2000; Eliuk & Makarov, 2015), means that today resolution of >150,000 can be attained providing increased mass accuracy, to around 1 ppm in even complex matrices (Bachi & Bonaldi, 2008). Predominantly, the domain of protein chemists initially, these high resolution hybrid instruments have now started to become commonplace in many analytical labs, and their capabilities for high resolution separation have been seized upon by those familiar with stable isotope methodologies.

### B. Isotope Incorporation Into OMICs Technologies

A vast amount of information has been accrued into the regulation of tissue protein turnover; however, this is a relatively crude process as tissues consist of tens of thousands of proteins that perform a variety of different functions and hence may, therefore, turnover at considerably different rates. Moreover, changes in the turnover of specific proteins may better reflect the underlying health and biology of a tissue or disease state than others, and may respond differently to specific interventions. Therefore, the ability to measure individual protein turnover rates accurately could be extremely informative, and may allow the potential development of dynamic biomarkers and diagnostics in the future.

Up until the early 2000s, proteomics was somewhat limited in the information it could provide, comparing the relative amounts of proteins under different static conditions. How, why, and at what rate these changes in protein expression were occurring was not possible to ascertain using classical proteomics. However, with the introduction of stable isotope tracers alongside classical proteomics, the dynamics of these changes in protein expression could finally be quantified (Pratt et al., 2002; Wu et al., 2004; McClatchy et al., 2007; Price et al., 2010). This was traditionally performed by providing deuterium labeled amino acids in culture medium to yeast cells or bacteria *in vitro* (Pratt et al., 2002), or ^15^N labeled algal cells to the diets of animals *in vivo* (McClatchy et al., 2007). By performing proteomics on different tissue/organs over a number of sampling time points, the incorporation of the labeled amino acids could be determined through the ratios of heavy to light peptides as measured using high resolution LC‐MS techniques, and hence rates of turnover of these individual proteins could be determined (Holmes et al., 2015). This was a major breakthrough, now not only could abundance of these proteins be determined but how rapidly they were turning over to produce these changes could be measured also.

While these techniques were ideally suited for pre‐clinical work, the ultimate goal would be to apply these methods in humans *in vivo*. The previously reported *in vitro* and animal work replaced amino acids in the diet with 100% labeled amino acids, something which is not possible for human work; therefore, an alternative method of labeling was devised using daily dosing with D_2_O (Price et al., 2012). Using this, the amino acids would become labeled with deuterium from D_2_O (via the route which has previously been described), the labeled amino acids are then incorporated into proteins over time. A process that had previously been validated in animal and cell culture models (Xiao et al., 2008; Rachdaoui et al., 2009; De Riva et al., 2010; Kasumov et al., 2011). By taking serial samples over time, Price was able to isolate peptides using LC‐MS/MS technology and extract mass isotopomer abundance data relating to 114 unique proteins. By measuring the increase in enrichment of peptides by deuterium over time and comparing to the initial unlabeled isotopomer ratio for these peptides, rates of turnover were kinetically modeled providing one of the first *in vivo* measures of the dynamics of the human proteome (Price et al., 2012). Using this technique, the turnover rates of a number of common proteins (such as plasma albumin) were validated showing good agreement with rates measured using this new D_2_O approach and rates previously reported using other AA tracer techniques (Price et al., 2012). Since this seminal paper, there has been a rapid uptake of this unique method in human research, and it is currently being used for drug discovery and in disease states (Li et al., 2012; McLaren et al., 2013; Wang et al., 2013; Decaris et al., 2014), while also being developed as minimally invasive tool for diagnostics and biomarker discovery (Holmes et al., 2015). This area seems ripe for exploitation using stable isotope tracers, despite initially being developed as a tool to monitor the basics of biological function; tracers have evolved as important tools with potential real life medical applications in diagnostics and drug pharmacology.

## V. CONCLUSIONS: WHAT DOES THE FUTURE HOLD?

Over a century has passed since that fateful, yet unbeknownst to him, hugely impactful discovery of the stable isotope of Neon by JJ Thomson (Thomson, 1913). Over the subsequent decades, analytical, industrial, and chemistry breakthroughs have provided the capacity for stable isotope techniques/approaches to become unparalleled tools, not only in the chemical and physical sciences, but also in the geological, physiological, and medical sciences. As such, stable isotopes can now provide us with information on things as diverse as geology (Menicucci, Matthews, & Spero, 2013), archaeology (Negash et al., 2015), environmental impact on ecosystems (Zhou et al., 2015b), the rates at which our body's build/breakdown tissues (Smith & Rennie, 1990), the effectiveness of a certain medical treatment or interventions (Schellekens et al., 2011), an individuals body composition (Kreisberg, Bowdoin, & Meador, 1970; Picou et al., 1976; Clark et al., 2014), the efficiency of our organs to function (Miele et al., 2013), and optimal nutrition for a healthy lifestyle (Rennie, 1999). Yet, this is only a snapshot of their potential uses, with future technical refinement and progress in MS development, there is no doubt that many more uses for these diverse tools will be in development.

So, where will stable isotope tracers take us in the future? As highlighted in the final sections of this review, in recent years, there has been an explosion in the use of OMICS‐technologies in the context of clinical prognostics, diagnostics, and biomarker development—not to mention biological insights in relation to understanding the underlying etiology of disease states. The emergence of metabolomics is still new compared to other “OMICS” fields, but improvements in analytical techniques and pattern recognition methods have contributed greatly to the increasing utility of metabolomics in clinical diagnostics. The feasibility of metabolomics for biomarker discovery is supported by the assumption metabolites are important players in biological systems and that diseases cause disruption of biochemical pathways, these are not new concepts. In fact, metabolomics has been shown to have benefits in various clinical areas. For example, clusters of metabolites have yielded prognostic and diagnostic biomarker value in relation to many diseases increasing in prevalence as a result of age (e.g., diabetes, CVD; Wang et al., 2011; Bhattacharya et al., 2014). As with the recent evolution of dynamic proteomics, metabolomics is seemingly following a similar path. While metabolomics in its standard form is an incredibly powerful tool with the potential for standalone prognostics and diagnostics, by itself it can only provide a static snapshot in time of relative changes in the metabolic profile in relation to a specific phenotype (Dunn et al., 2011, 2015). However, with the inclusion of stable isotope tracers, the opportunity to quantify the rates and direction of changes in these profiles, alongside the temporal nature of this change as well as the rate of flux through associated metabolic pathways, something which is being termed “fluxomics,” becomes possible (Winter and Kromer, 2013; Niedenfuhr et al., 2015; Overmyer et al., 2015). The benefits of such newly developed techniques have been highlighted in the field of drug development and cancer research, where the use of stable isotope‐resolved metabolomics (SIRM) have provided information on the distinct metabolic traits of cancer tissues compared to non‐cancerous tissues (Fan et al., 2009), which could in future be used to aid development of therapeutic interventions to target this altered metabolism in cancer cells (for more information on this the reader is guided to Lane et al. (2011); Fan et al. (2012)). It is these dynamic OMICs techniques that will no doubt flourish in forthcoming years, with their potential as diagnostic biomarkers a key application. Indeed, this is already becoming apparent with industry dedicating extensive resources toward developing these approaches. While stable isotope tracers will remain a cornerstone in their traditional roles investigating basic biology and metabolism, the continued development of technologies in the MS fields will continue to push forward new and novel applications for stable isotope tracers in the future and it is clear to see that these tools will remain vital to many of us in the scientific community for many years to come.

**ABBREVIATIONS**
AA: amino acid
APCI: atmospheric pressure chemical ionization
APE: atom percent excess
BCAA: branched chain AA
CI: chemical ionization
EAA: essential AA
EI: electron ionization
ESI: electrospray ionization
FT: fourier transform
GC: gas chromatography
GC‐C‐IRMS: gas chromatography‐combustion‐IRMS
GC‐Pyrolysis‐IRMS: gas chromatography‐pyrolysis‐ IRMS
GLC: gas–liquid chromatography
ig: intragastric
IRMS: isotope ratio mass spectrometer
iv: intravenous
LC: liquid chromatography
LC‐MS/MS: LC‐tandem mass spectrometer
MALDI: matrix‐assisted laser desorption ionisation
MPB: muscle protein breakdown
MPS: muscle protein synthesis
MRM: multiple reaction monitoring
MS: mass spectrometer
m/z: mass‐charge ratio
QIT: quadrupole ion trap
QQQ: triple quadrupole
QTOF: quadrupole time of flight
SRM: single reaction monitoring
TOF: time of flight
